# Supplementary material for: Tead1a Initiates Transcriptional Priming Through the TEAD1a/YAP‐Notch1‐Spi1/Cebpα Axis to Promote Neutrophil Fate
Source: Adv Sci (Weinh). 2025 Aug 28;12(41):e05441. doi: 10.1002/advs.202505441 (PMC12591127; doi:10.1002/advs.202505441)
Supplement: Supplementary file 4 — Supplementary Dataset [file ADVS-12-e05441-s004.zip › 3-6.zip!/3-6/02-6.cluster/cluster16/Enrich/ego_kegg.pdf]

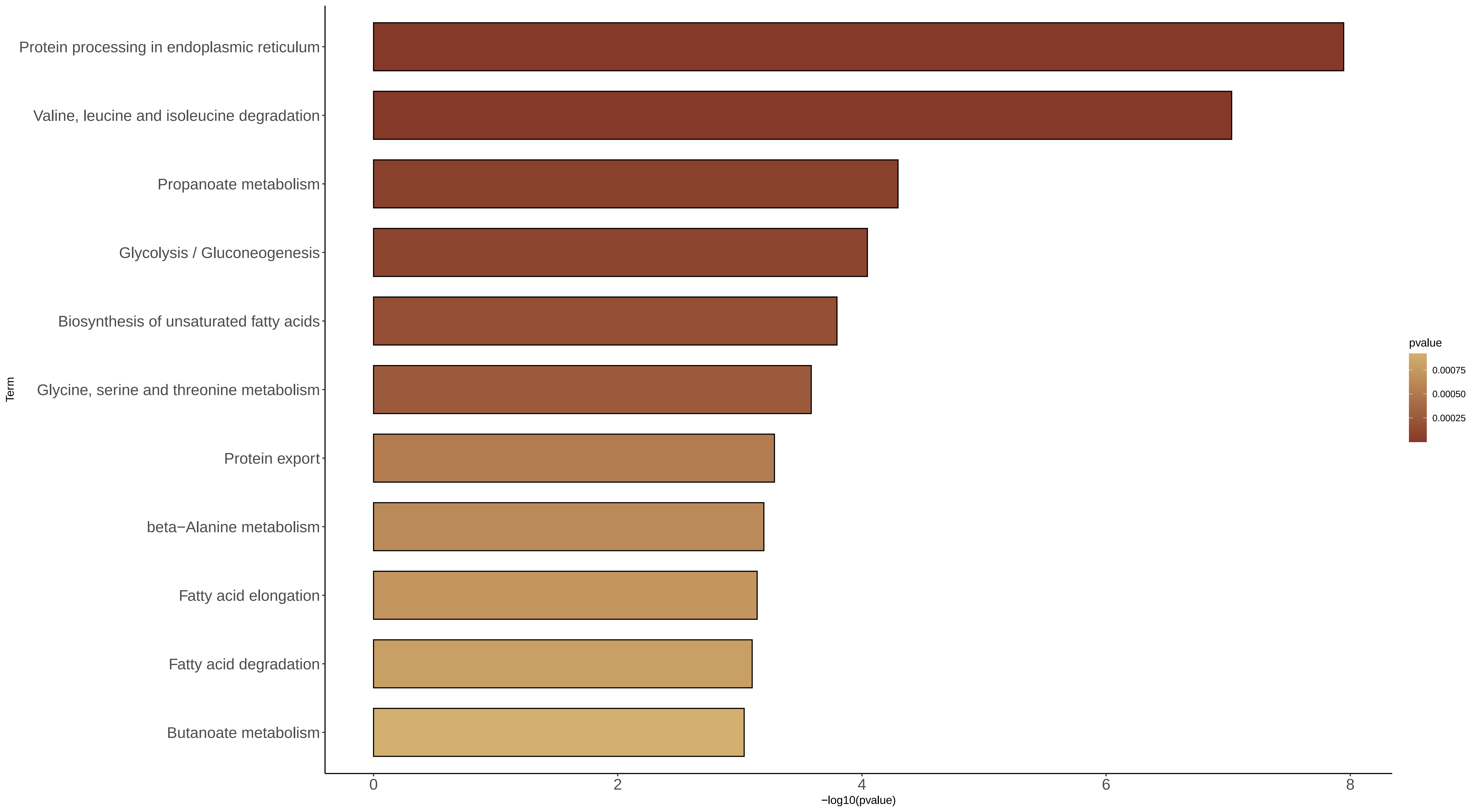
Protein processing in endoplasmic reticulum
Valine, leucine and isoleucine degradation
Propanoate metabolism
Glycolysis / Gluconeogenesis
Biosynthesis of unsaturated fatty acids
Glycine, serine and threonine metabolism
Protein export
beta−Alanine metabolism
Fatty acid elongation
Fatty acid degradation
Butanoate metabolism
Term
0
2
4
6
8
−log10(pvalue)
pvalue
0.00075
0.00050
0.00025
